# Myocardial blood flow reference values for 13N-ammonia PET myocardial perfusion imaging in patients without flow-limiting coronary artery disease

**DOI:** 10.1007/s00259-025-07196-0

**Published:** 2025-03-14

**Authors:** Rita Pingree, Susanne Markendorf, Dimitrios Moysidis, Christoph Ryffel, Magdalena Stuetz, Raffael Ghenzi, Marko Gajic, Dominik C. Benz, Aju P. Pazhenkottil, Andreas A. Giannopoulos, Philipp A. Kaufmann, Simon Winther, Ronny R. Buechel

**Affiliations:** 1 Department of Nuclear Medicine, Cardiac Imaging, University Hospital and University of Zurich, Zurich, Switzerland; 2 Department of Cardiology, Gødstrup Hospital, Herning, Denmark

**Keywords:** Positron emission tomography, Myocardial flow reserve, Myocardial blood flow, Reference values, Normal values

## Abstract

**Purpose:**

To determine the most important patient factors influencing quantitative MBF and to report the lower (LRL) and upper (URL) reference limits for 13N-ammonia positron emission tomography (PET) myocardial perfusion imaging (MPI).

**Methods:**

Patients who underwent 13N-ammonia PET-MPI were screened, and those with evidence of myocardial ischemia or scar, known cardiomyopathy, impaired left ventricular function, non-response to vasodilators, and those who underwent a stress-rest protocol were excluded. Multiple linear regression analyses were performed to identify independent predictors of rest MBF (rMBF), stress MBF (sMBF), and myocardial flow reserve (MFR), and predictor importance was calculated. Finally, median, LRL, and URL for rMBF, sMBF, and MFR were calculated based on the presence of predictors.

**Results:**

Among 784 patients with a median coronary artery calcium score (CACS) of 69, median rMBF was 0.75mL∙min^− 1^∙g^− 1^ (LRL = 0.49 mL∙min^− 1^∙g^− 1^; URL = 1.33 mL∙min^− 1^∙g^− 1^), median sMBF was 2.41mL∙min^− 1^∙g^− 1^ (LRL = 1.42 mL∙min^− 1^∙g^− 1^; URL = 3.73 mL∙min^− 1^∙g^− 1^), and median MFR was 3.09 (LRL = 2.11; URL = 4.65). The body mass index (BMI) was the single most important independent predictor of rMBF, sMBF, and MFR (predictor importance of 72%, 87%, and 41%, respectively; standardized β=-0.434, -0.566 and − 0.174, respectively). Additional predictors were sex and hypertension for rMBF, sex for sMBF, and hypertension and CACS for MFR.

**Conclusion:**

In patients without flow-limiting CAD, MBF is strongly influenced by the patient’s habitus, whereby median and reference limits for sMBF and rMBF decrease with increasing BMI. Consequently, MFR exhibits stable lower reference limits across a wide range of BMI and may be considered the most robust quantitative parameter derived from 13N-ammonia PET-MPI.

**Supplementary Information:**

The online version contains supplementary material available at 10.1007/s00259-025-07196-0.

## Graphic Abstract

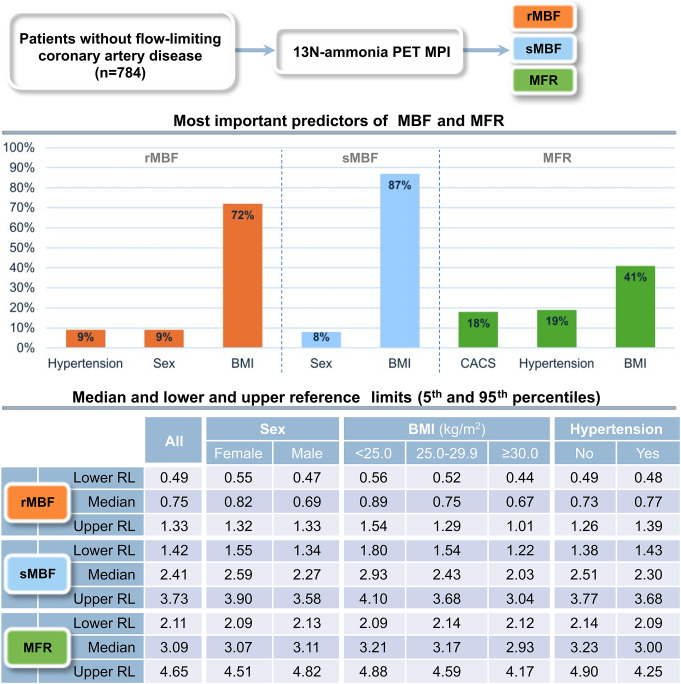

## Introduction

Positron emission tomography (PET) myocardial perfusion imaging (MPI) is a valuable non-invasive imaging technique used to detect ischemic heart disease and to evaluate myocardial blood flow quantitatively. This modality leverages radiotracers such as O15-water, Rubidium-82, or 13N-ammonia to provide high-resolution images of myocardial perfusion under rest and stress conditions, thereby providing superior diagnostic performance compared to other non-invasive ischemia testing [1]. PET MPI not only facilitates the identification of regional perfusion abnormalities indicative of flow-limiting coronary artery disease (CAD) but also allows for absolute quantification of myocardial blood flow at rest (rMBF) and during pharmacological stress (sMBF), enabling the calculation of the myocardial flow reserve (MFR) and an assessment of microvascular function. It allows comprehensive risk stratification, aids clinical decision-making, and improves clinical outcomes in cardiovascular care [2–5].

Several radiotracers are used in PET for the assessment of myocardial perfusion. While O15-water is considered the most accurate radiotracer for quantifying MBF, its use is limited in clinical practice due to its short half-life requiring an on-site cyclotron and lack of approval for clinical use in the U.S. and most European countries. The use of Rubidium-82, on the other hand, is more widespread due to the possibility of generator-based production, but this radiotracer exhibits limitations regarding spatial resolution and non-linear extraction, leading to an underestimation of stress MBF. By contrast, 13N-ammonia maintains a nearly linear extraction fraction, especially under hyperemic conditions, which improves its utility in assessing sMBF and MFR [6, 7].

Routine absolute flow quantification is arguably PET’s most significant unique advantage compared to other non-invasive imaging techniques. However, while extensive data on cutoff values for MFR and MBF for identifying ischemia and predicting prognosis are available, information on normal reference values and the physiological and patient-specific factors influencing them is lacking in the published literature. Upon our review, only a single recent study has reported normal values for Rubidium-82 PET/computed tomography (CT) [7], and there are currently no comparable studies for 13N-ammonia.

The present study investigates physiological factors influencing rMBF, sMBF, and MFR in patients without flow-limiting CAD and establishes reference values for quantitative parameters derived from 13N-ammonia PET MPI.

## Methods

### Patient population

This is a retrospective single-center study with patients who underwent 13N-ammonia PET/CT MPI scans for the assessment of suspected chronic coronary syndrome between December 2014 and June 2023. From the hospital’s electronic health records, all patients were screened who exhibited no regional perfusion defects (i.e., normal stress and rest retention images) and without any evidence of global ischemia (i.e., with global sMBF ≤ 1.99 mL/min and global MFR ≤ 2.12) [8]. Furthermore, patients suspected to be non-responders to adenosine or regadenoson (i.e., with a global MFR < 1.5) [9] or those stressed with dobutamine were excluded. Finally, pre-defined exclusion criteria included all patients with known or suspected cardiomyopathy or a global left ventricular ejection fraction (LVEF) < 50%. The local ethics committee approved the study (BASEC-Nr. 2023 − 01220) and granted an exemption for the need for written informed consent in those patients who did not explicitly refuse to use their personal data for research.

## PET acquisition, reconstruction, and analysis

All patients underwent clinically indicated PET MPI using 13N-ammonia acquired at rest and during pharmacological stress (adenosine infused at 0.14 mg ∙ kg^− 1^ ∙ min^− 1^ over 6 min or a single bolus injection of 400 µg of regadenosone). As previously reported, all image data were acquired in list mode on a PET/CT scanner (Discovery MI, Discovery VCT, and Discovery DST, all GE Healthcare, Waukesha, WI, USA) [10].

In brief, a body mass index (BMI)-adapted dose of 13N-ammonia was injected (i.e., 266 ± 68 and 424 ± 118 Megabecquerels at rest and during stress, respectively). The time interval between rest and stress image acquisition was 24 ± 5 min. Datasets were reconstructed using ordered subset expectation maximization (OSEM, VUE Point HD or VUE Point FX, two iterations, 16 subsets), and a 5 mm Hanning filter and standard decay, scatter, and sensitivity corrections (voxel size 2.34, 2.34, 2.80–3.27) were applied. Non-contrast CT was used for attenuation correction of PET/CT datasets. Additional non-contrast electrocardiogram (ECG)-triggered CT scans were acquired to calculate the coronary artery calcium score (CACS) in all patients. PET image acquisition was acquired in list mode over 14 min. For both stress and rest, dynamic datasets were reconstructed from the first 7 min of acquisition and consisted of 9 frames of 10 s duration, 6 frames of 15 s, 3 frames of 20 s, 2 frames of 30 s, and 1 frame of 120 s. Static and ECG-gated datasets were reconstructed from the last 10 min of the acquisition. MBF during stress (sMBF), at rest (rMBF_uncorrected_ and rMBF, corrected for workload using the rate-pressure product [RPP]), MFR, and LVEF at rest were calculated using commercially available software (QPET 2017.7 Cedars-Sinai Medical Center, Los Angeles, CA, USA). Finally, an index of coronary vascular resistance (CVR) was determined as the ratio of mean arterial blood pressure (MAP, calculated as [diastolic BP + (0.3 ∙ (systolic BP– diastolic BP]) to rMBF (rCVR) and sMBF (sCVR) [11].

### Statistical analysis

Statistical analysis was performed using SPSS (version 29.0, IBM Corporation, Armonk, NY, USA) and MedCalc (version 19.6.4, MedCalc Software Ltd., Ostend, Belgium). Normally distributed continuous variables are expressed as mean ± standard deviation. Otherwise, the median and interquartile range (IQR; 25th to 75th percentile) are given. Spearman correlation analysis was applied for non-normally distributed variables. Categorical variables are represented as absolute numbers and percentages. Unpaired T-tests were used to compare normally distributed continuous variables. The chi-square test was used to compare dichotomous variables. The Mann–Whitney U test was applied for non-normally distributed variables, and the Jonckheere-Terpstra test for ordered alternatives where the independent variable was of an ordinal type (i.e., BMI groups) with pairwise-comparison and post-hoc Bonferroni correction. Multiple linear regression with post-hoc Bonferroni correction was used to identify possible predictors of rMBF, sMBF, and MFR out of the following candidate variables: age, sex, BMI, type of vasodilator, CACS, LVEF, and the cardiovascular risk factors of smoking, hypertension, dyslipidemia, and diabetes. Tests for excluding the presence of multicollinearity were performed, and the Durbin-Watson statistical test was applied to test for autocorrelation. Standardized coefficients (β) and the relative importance of potential predictors are provided. A supplementary and extended multiple linear regression analysis that includes cardiac medication was also performed. To assess reference values, we calculated the upper and lower reference limits for rMBF, sMBF, and MFR. Therefore, the 5th percentile for the left-sided reference interval and the 95th percentile for the right-sided reference intervals were calculated, following the Clinical and Laboratory Standards Institute (CLSI) guidelines. Finally, to ensure the absence of correlation between BMI and radiotracer activity acquired from within the LV myocardium during PET imaging, the summed acquired left ventricular myocardium activity (in Becquerels [Bq] per Milliliter [mL]) in a random selection of 15% (*n* = 118) of all subjects across all tertiles of BMI was recorded. Finally, for all patients with symptoms, we calculated the CACS-weighted clinical likelihood for obstructive CAD [12]. The latter model considers symptoms, traditional cardiovascular risk factors, and CACS. It has been shown to be superior to previous models, and its use is recommended by the current ESC guidelines for the management of chronic coronary syndromes [5]. All statistical tests were 2-tailed, and a p-value of less than 0.05 was considered statistically significant.

## Results

### Study population

**Fig. 1:**
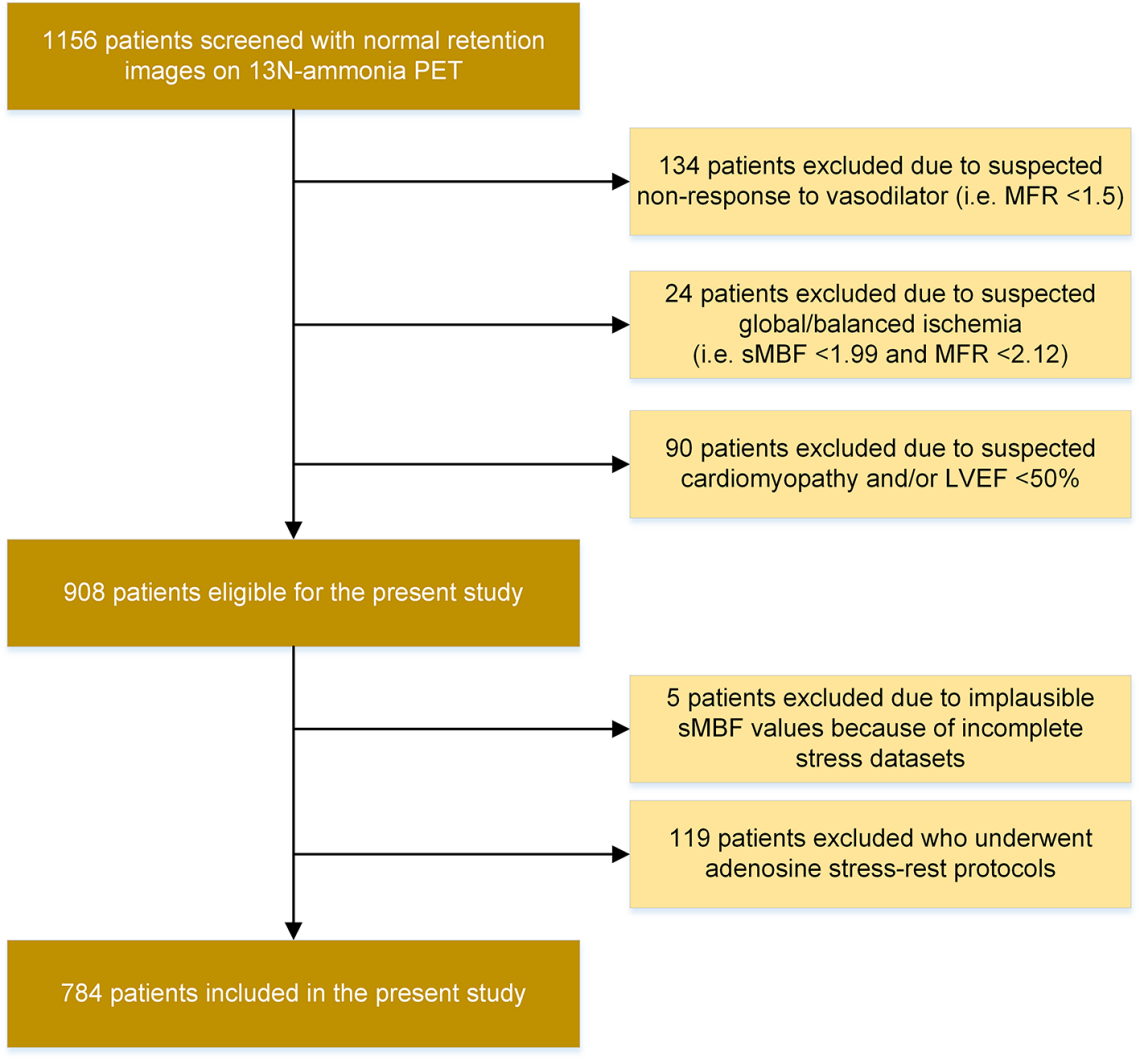
Consolidated standards of reporting trials diagram of patient enrollment

A total of 784 patients were included in the final analysis. Figure 1 depicts the diagram of patient enrollment. Out of a total of 908 patients who did not exhibit any pre-defined exclusion criteria, five patients had corrupted and/or incomplete stress datasets. Additionally, 119 patients were excluded post hoc because they underwent a rapid adenosine stress-rest protocol, which was recently discovered to cause high rMBF values because of a prolonged effect of adenosine [13]. Therefore, these patients were excluded to avoid protocol-induced confounding.

Table 1 exhibits the demographics and characteristics of the overall population and is stratified according to sex. Slightly more than half of the patients (54.5%) underwent PET MPI because of chest pain or dyspnea. In comparison, the indication for PET MPI in the remaining patients (45.5%) was due to unspecific symptoms (e.g., palpitations, syncope, fatigue etc.) or incidental findings during a checkup or pre-surgical examination (e.g., pathological ECG or echocardiography, coronary calcifications in chest imaging etc.) in patients without specific cardiac symptoms. The majority of patients exhibited a low or low-to-moderate clinical likelihood of having obstructive CAD, as defined by the coronary artery calcium score-weighted model, with female patients having a lower likelihood than male patients.

**Table 1:** Baseline characteristics in the overall population and stratified by sex

		Sex		
	All patients (*n* = 784)	Female (*n* = 315)	Male (*n* = 469)	p-value
Age (years)	63 ± 13	65 ± 12	61 ± 13	**< 0.001**
Female sex	315 (40.2)			
Body mass index (kg/m^2^)	28.9 ± 7.1	29.2 ± 7.9	28.7 ± 6.5	0.357
Indication for PET MPI				
Typical angina	105 (13.4)	57 (18.1)	48 (10.2)	**0.002**
Atypical angina	179 (22.8)	93 (29.5)	86 (18.3)	**< 0.001**
Non-anginal chest pain	41 (5.2)	18 (5.7)	23 (4.9)	0.627
Dyspnea	103 (13.1)	52 (16.5)	51 (10.9)	**0.024**
Other	356 (45.5)	96 (30.5)	260 (55.4)	**< 0.001**
Cardiovascular risk factors				
Hypertension	400 (51.0)	159 (50.5)	241 (51.4)	0.827
Dyslipidemia	293 (37.4)	134 (42.5)	159 (33.9)	**0.016**
Diabetes	180 (23.0)	63 (20.0)	117 (24.9)	0.119
Positive family history of CAD	114 (14.5)	59 (18.7)	55 (11.7)	**0.007**
Smoking	177 (22.6)	54 (17.1)	123 (26.2)	**0.003**
Cardiac medication				
Antithrombotics	125 (15.9)	51 (16.2)	74 (15.8)	0.921
Beta-blockers	137 (17.5)	57 (18.1)	80 (17.1)	0.703
ACEI/ARB	212 (27.0)	81 (25.7)	131 (27.9)	0.513
Lipid-lowering drugs	187 (23.9)	76 (24.1)	111 (23.7)	0.932
Hemodynamic parameters at rest				
Heart rate (bpm)	68 [60–77]	69 [61–79]	68 [60–75]	**0.020**
Systolic blood pressure (mmHg)	137 [123–152]	141 [128–155]	133 [121–150]	**< 0.001**
Diastolic blood pressure (mmHg)	77 [69–85]	76 [68–83]	78 [70–87]	**0.003**
RPP (mmHg ∙ bpm)	9,375 [7696–11211]	9,620 [8255–11506]	9,159 [7500–10790]	**< 0.001**
Hemodynamic parameters during vasodilation				
Heart rate (bpm)	87 [77–98]	92 [84–103]	84 [75–94]	**< 0.001**
Systolic blood pressure (mmHg)	133 [118–150]	140 [121–154]	129 [116–146]	**< 0.001**
Diastolic blood pressure (mmHg)	75 [66–84]	75 [66–83]	75 [65–85]	0.813
RPP (mmHg ∙ bpm)	11,603 [9513–13985]	12,690 [10296–15360]	10,900 [9052–13113]	**< 0.001**
Imaging findings				
LVEF (%)	67 ± 8	70 ± 7	64 ± 7	**< 0.001**
CACS	69 [1-337]	33 [0-191]	103 [5-522]	**< 0.001**
CACS-weighted clinical likelihood of obstructive CAD (%)^*^	13.9 [4.0-29.3]	6.9 [2.3–18.5]	20.0 [8.0-36.6]	**< 0.001**
Very low (≤ 5%)	137 (32.0)	99 (43.8)	38 (18.8)	**< 0.001**
Low (6–15%)	104 (24.3)	56 (24.8)	48 (23.8)	0.503
Low-to-moderate (16–50%)	159 (37.1)	70 (31.0)	89 (44.1)	**0.023**
Moderate-to-high (51–85%)	28 (6.5)	1 (0.4)	27 (13.4)	**< 0.001**

Values are mean ± SD, absolute numbers and percentages in parentheses or median, and IQR in brackets

* Pre-test probabilities were calculated for symptomatic patients only (*n* = 428; female *n* = 226, male *n* = 202)

*RPP = rate pressure product; ACEI = angiotensin-converting enzyme inhibitor; ARB = angiotensin receptor blocker; LVEF = left ventricular ejection fraction; CACS = coronary artery calcium score*

### Predictors of quantitative MBF measurements

**Fig. 2:**
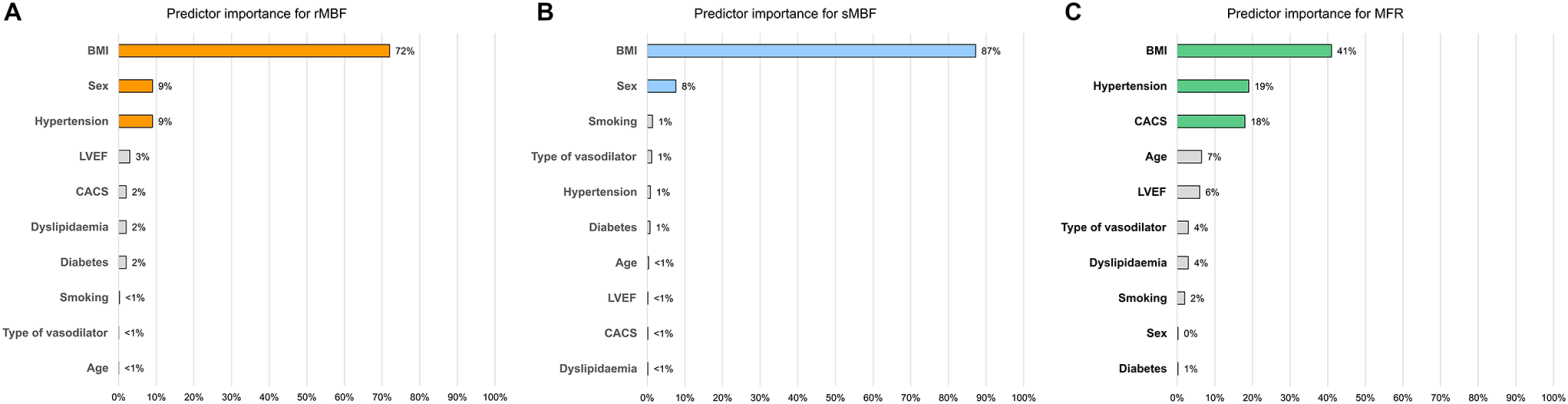
Predictor importance for MBF. The relative importance of each predictor in estimating the prediction model for rMBF (**A**), sMBF (**B**), and MFR (**C**) is provided. Statistically non-significant predictors are greyed out

Multiple regression analysis was run to identify possible predictors of rMBF. The model statistically significantly predicted rMBF F(10,773) = 27.139, adjusted r^2^ = 0.251, *p* < 0.001). Among all variables, BMI (β = -0.434, *p* < 0.001, sex (β = -0.171, *p* < 0.001), and hypertension (β = 0.162, *p* < 0.001) added independently and statistically significantly to the prediction. Figure 2A provides an overview of the importance of the predictors.

Similarly, for sMBF, multiple regression analysis, including the same variables, revealed that the model statistically significantly predicted sMBF F(10,773) = 46.673, adjusted r^2^ = 0.368, *p* < 0.001). Among all variables, only BMI (β = -0.566, *p* < 0.001) and sex (β = − 0.187, *p* < 0.001) added independently and statistically significantly to the prediction. Figure 2B provides an overview of the importance of the predictors.

Finally, multiple regression analysis with the same variables for MFR revealed that the model weakly but statistically significantly predicted MFR F(10,773) = 7.488, adjusted r^2^ = 0.077, *p* < 0.001). Among all variables, BMI (β = -0.174, *p* < 0.001), hypertension (β = -0.121, *p* = 0.001), and CACS (β = -0.120, *p* = 0.001) added independently and statistically significantly to the prediction. Figure 2C provides an overview of the importance of the predictors.

Durban-Watsons statistics for all regression analyses did not indicate any autocorrelation (with values ranging between 1.805 and 2.011), and all tests for multicollinearity were negative.

Among all variables, only BMI consistently and significantly predicted all MBF parameters. By contrast, female sex increased both rMBF and sMBF, while MFR was not impacted. CACS predicted MFR only, while hypertension impacted rMBF and MFR but not sMBF.

Of note, no correlation was found between BMI and count rates during rest and stress PET imaging (*r* = -0.073, *p* = 0.433 and *r* = 0.065, *p* = 0.483, respectively; supplementary Figure S1).

The results of a more extensive analysis, including medication, are provided in the supplementary results. Notably, neither beta-blockers nor antithrombotics nor antihypertensive medication or lipid-lowering drugs were statistically significant independent predictors for rMBF, sMBF, or MFR (Figure S2).

## Distribution of MBF parameters and reference limits

Based on the significant independent predictors identified by the prior regression analyses, a detailed analysis of the distribution and impact of these predictors on MBF was performed and the upper and lower reference values of normal are provided. The following results are based on rMBF and MFR corrected for the RPP. Values not corrected for RPP (i.e., rMBF_uncorrected_ and MFR_uncorrected_) are provided in the supplementary tables (Tables S1-S6).

Table 2 depicts MBF parameters in the overall population and stratified by sex. Of note, both median rMBF and sMBF values were higher in female than male subsets (*p* < 0.001), resulting in a comparable MFR. Consequently, upper and lower reference limits for rMBF and sMBF were generally higher in female than male subsets but similar for MFR.

**Table 2:** Myocardial flow parameters in the overall population and stratified by sex

		Sex		
	All patients (*n* = 784)	Female (*n* = 315)	Male (*n* = 469)	p-value
rMBF (ml ∙ min^− 1^ ∙ g^− 1^) Lower reference limit Upper reference limit	0.75 [0.62–0.94] 0.49 1.33	0.82 [0.69–0.99] 0.55 1.32	0.69 [0.58–0.87] 0.47 1.33	**< 0.001**
sMBF (ml ∙ min^− 1^ ∙ g^− 1^) Lower reference limit Upper reference limit	2.41 [1.97–2.94] 1.42 3.73	2.59 [2.07–3.10] 1.55 3.90	2.27 [1.89–2.80] 1.34 3.58	**< 0.001**
MFR Lower reference limit Upper reference limit	3.09 [2.56–3.67] 2.11 4.65	3.07 [2.62–3.59] 2.09 4.51	3.11 [2.54–3.74] 2.13 4.82	0.457

Values given are median and IQR in brackets and lower (i.e., 5th percentile) and upper (i.e., 95th percentile) reference limits

Table 3 depicts MBF parameters across different BMI groups. As expected from the regression analysis, there was a substantial and statistically significant decrease in median rMBF and sMBF with increasing BMI. The decrease was slightly more pronounced for sMBF, resulting in a consequent decrease of MFR with increasing BMI groups, and pairwise comparison revealed significant differences in MFR between patients in the highest vs. the lowest and the middle BMI groups.

**Table 3:** Myocardial flow parameters stratified by BMI groups

	BMI (kg/m^2^)			
	< 25.0 (*n* = 278)	25.0-29.9 (*n* = 214)	≥ 30.0 (*n* = 292)	p-value
rMBF (ml ∙ min^− 1^ ∙ g^− 1^) Lower reference limit Upper reference limit	0.89 [0.72–1.07] 0.56 1.54	0.75 [0.62–0.90] † 0.52 1.29	0.67 [0.57–0.79] ^*^† 0.44 1.01	**< 0.001**
sMBF (ml ∙ min^− 1^ ∙ g^− 1^) Lower reference limit Upper reference limit	2.93 [1.80–3.36] 1.80 4.10	2.43 [2.04–2.84] † 1.54 3.68	2.03 [1.64–2.39] ^*^† 1.22 3.04	**< 0.001**
MFR Lower reference limit Upper reference limit	3.21 [2.67–3.84] 2.09 4.88	3.17 [2.63–3.81] 2.14 4.59	2.93 [2.47–3.44] ^*^† 2.12 4.17	**< 0.001**

Values given are median and IQR in brackets and lower (i.e., 5th percentile) and upper (i.e., 95th percentile) reference limits

* *p* < 0.001 vs. BMI 25.0–29.9 kg/m^2^

† *p* < 0.001 vs. BMI < 25.0 kg/m^2^

Table 4 provides a more detailed analysis through stratification by sex and BMI groups. Of note, the relative decrease with increasing BMI groups was comparable between males and females for rMBF (-23% and -26% vs. the lowest BMI group, respectively), sMBF (-29% and -30%, respectively), and MFR (-9% and -7%), with females generally exhibiting higher absolute rMBF and sMBF values, thus resulting in comparable MFR values and lower reference limits among BMI groups.

**Table 4:** Myocardial flow parameters stratified by sex and BMI groups

	Female				Male			
		rMBF (ml ∙ min^− 1^ ∙ g^− 1^)	sMBF (ml ∙ min^− 1^ ∙ g^− 1^)	MFR		rMBF (ml ∙ min^− 1^ ∙ g^− 1^)	sMBF (ml ∙ min^− 1^ ∙ g^− 1^)	MFR
BMI (kg/m^2^)	n				n			
< 25.0	121	0.94 [0.79–1.08] 0.63 1.57	3.04 [2.60–3.50] 1.86 4.27	3.22 [2.82–3.79] 2.09 4.65	157	0.83 [0.65–1.06] 0.53 1.54	2.75 [2.32–3.24] 1.76 3.91	3.20 [2.54–3.89] 2.07 5.03
25.0-29.9	61	0.85 [0.71–1.04] 0.57 1.46	2.65 [2.36–3.03] 1.69 3.91	3.15 [2.64–3.78] 2.14 4.28	153	0.71 [0.60–0.86] 0.50 1.23	2.28 [1.99–2.76] 1.51 3.56	3.17 [2.63–3.82] 2.12 4.79
≥ 30.0	133	0.73 [0.62–0.84] 0.50 1.03	2.11 [1.79–2.53] 1.32 3.27	2.88 [2.46–3.43] 2.06 4.27	159	0.63 [0.54–0.73] 0.39 1.00	1.90 [1.53–2.20] 1.11 2.87	2.94 [2.48–3.45] 2.18 4.06

Values given are median and IQR in brackets and lower (i.e., 5th percentile) and upper (i.e., 95th percentile) reference limits

Table 5 details MBF parameters stratified by sex and the presence or absence of hypertension. Of note, differences in MBF and MFR in patients with vs. without hypertension MFR were mainly present in the male subset, while the parameters did not differ between both groups in the female subset.

**Table 5:** Myocardial flow parameters in patients with versus without hypertension in the overall population and stratified by sex

	Overall population			Female			Male		
	No hypertension (*n* = 384)	Hypertension (*n* = 400)	p-value	No hypertension (*n* = 156)	Hypertension (*n* = 159)	p-value	No hypertension (*n* = 228)	Hypertension (*n* = 241)	p-value
rMBF (ml ∙ min^− 1^ ∙ g^− 1^) Lower reference limit Upper reference limit	0.73 [0.60–0.92] 0.49 1.26	0.77 [0.63–0.95] 0.48 1.39	**0.037**	0.82 [0.69–0.99] 0.55 1.30	0.82 [0.69-1.00] 0.53 1.43	0.648	0.68 [0.58–0.83] 0.46 1.25	0.74 [0.60–0.92] 0.47 1.39	**0.021**
sMBF (ml ∙ min^− 1^ ∙ g^− 1^) Lower reference limit Upper reference limit	2.51 [2.03–2.99] 1.38 3.77	2.30 [1.93–2.91] 1.43 3.68	**0.016**	2.68 [2.09–3.13] 1.52 3.92	2.52 [2.07–3.08] 1.56 3.90	0.403	2.39 [1.97–2.85] 1.30 3.68	2.15 [1.84–2.77] 1.34 3.52	**0.011**
MFR Lower reference limit Upper reference limit	3.23 [2.65–3.85] 2.14 4.90	3.00 [2.51–3.52] 2.09 4.25	**< 0.001**	3.06 [2.64–3.62] 2.09 4.61	3.08 [2.55–3.52] 2.09 4.28	0.419	3.35 [2.70–3.95] 2.29 5.05	2.92 [2.44–3.50] 2.09 4.23	**< 0.001**

Values given are median and IQR in brackets and lower (i.e., 5th percentile) and upper (i.e., 95th percentile) reference limits

Table 6 provides details on MBF parameters stratified by CACS groups. Interestingly, patients with a CACS > 0 exhibited higher rMBF but lower sMBF and MFR values than those with CACS = 0. There were no differences among groups in a subanalysis confined to patients with CACS > 0 (p-values ranging from 0.065 to 0.705), with comparable results if the analysis was performed separately for male and female subsets.

**Table 6:** Myocardial flow parameters stratified by CACS groups

	CACS				
	0 (*n* = 182)	1–99 (*n* = 246)	100–399 (*n* = 171)	> 400 (*n* = 185)	p-value
rMBF (ml ∙ min^− 1^ ∙ g^− 1^) Lower reference limit Upper reference limit	0.72 [0.60–0.86] 0.48 1.32	0.74 [0.61–0.91] 0.47 1.29	0.79 [0.65–0.97] ^*^ 0.51 1.33	0.78 [0.63–0.98] 0.49 1.37	**0.006**
sMBF (ml ∙ min^− 1^ ∙ g^− 1^) Lower reference limit Upper reference limit	2.59 [2.05–3.05] 1.49 3.96	2.38 [1.82–2.96] ^*^ 1.33 3.57	2.38 [2.03–2.92] 1.54 3.90	2.29 [1.86–2.78] ^*^ 1.36 3.61	**0.023**
MFR Lower reference limit Upper reference limit	3.45 [2.75–4.01] 2.14 5.02	3.09 [2.50–3.65] † 2.05 4.51	3.06 [2.65–3.61] † 2.16 4.41	2.88 [2.47–3.29] † 2.16 4.13	**< 0.001**

Values given are median and IQR in brackets and lower (i.e., 5th percentile) and upper (i.e., 95th percentile) reference limits

* *p* < 0.05 vs. CACS 0

† *p* < 0.005 vs. CACS 0

Although age was not per se an independent predictor of MBF or MFR, values for different age groups may be of interest and are provided in a supplementary table (Table S7). Of note, sMBF was not significantly related to age (*r* = 0.051, *p* = 0.150), while there was a weak correlation between age and rMBF (*r* = 0.187, *p* < 0.001) and, consequently, inversely between age and MFR (*r* = -0.116, *p* = 0.001). Importantly, however, the lower reference values for MFR did not differ substantially across age groups.

Finally, median and reference values for sCVR and rCVR are also provided in the supplementary Table S8.

## Discussion

The current study aims to provide insights into the interplay of various individual factors and MBF parameters in patients without flow-limiting CAD and establish how these parameters modify and impact expected normal reference values.

First, the results indicate that body habitus, as reflected by BMI, has the most profound impact on MBF, accounting for more than two-thirds of the relative importance of all potential predictors investigated. However, BMI seems to affect rMBF and sMBF similarly, thereby limiting the impact on MFR. Second, sMBF and rMBF differ between male and female subsets, with the former exhibiting higher values. However, as the relative differences were again almost identical for both rMBF and sMBF, median MFR did not differ significantly. More importantly, the lower reference limit for MFR was nearly identical between both sexes. Third, as the same trends were seen in the analysis of different BMI groups, this study supports the reliability of MFR for clinical routine. By contrast, the results suggest that reference limits for sMBF should be applied cautiously in clinical routine, as these can vary substantially depending on the individual body habitus. Hence, BMI should be taken into consideration. However, the results do not indicate that the body habitus has any different effects in male or female subsets, as the relative decrease of all parameters with increasing BMI was comparable between both sexes.

Furthermore, the risk factor of hypertension affected MBF parameters. Hypertension led to slightly higher rMBF values but lower sMBF values, resulting in a lower median MFR but, importantly, nearly identical lower reference limits for MFR. The results also indicate that hypertension may have a sex-specific impact because the differences were mainly driven by differences among MBF parameters in the male subset but not the female subset.

Considering CACS, patients with any calcifications tended to exhibit higher rMBF but lower sMBF and MFR values than those without any evidence of coronary calcifications. A theoretical explanation for this finding is that a higher prevalence of (early or *forme fruste*) microvascular dysfunction in patients with coronary calcifications could at least partly explain this difference for sMBF. Overall, we found only a weak inverse correlation between CACS and MFR. Importantly, as with other factors, the lower limits of reference for MFR remained relatively stable with increasing CACS, as opposed to sMBF reference values.

The results presented here are particularly significant given the relatively limited evidence available for establishing reference values for the various tracers used in PET MPI. While the vast majority of prior publications have mainly focused on establishing MBF cutoff values for detecting myocardial ischemia or predicting future adverse cardiovascular events [2–4, 8, 14–18], only a few have reported on normal or reference values in patients without obstructive or flow-limiting CAD.

A few historical publications have reported normal MBF values for 13N-ammonia [6, 7], Rubidium-82 [6], and 15O-water [19]. However, these studies were very small (*n* ≤ 56) and performed in young and healthy volunteers, rendering comparability with the present study difficult, if not impracticable. Studies utilizing healthy volunteers are important to study the fundamental physiological aspects of MBF and its regulating impactors. In clinical practice, however, these values are of limited use as a reference, as the population characteristics between healthy volunteers and the typical population referred for PET MPI differ fundamentally. Against this background, this study focused on the assessment and reporting of reference values obtained from a cohort most likely compatible with a “healthy” population that is more likely to be encountered in daily clinical routine.

A recent study by Sperry et al. aimed to provide age- and sex-specific reference values obtained from Rubidium-82 PET MPI in a large population of 2789 patients [20]. The authors of this study are to be commended for attempting to add important additional information to the sparse existing literature. However, the study design raises some concerns. Sperry et al. decided to exclude all patients with a CACS > 0 as well as those with diabetes or renal failure. While this approach may arguably lead to a more “healthy” population, the latter is not likely to reflect the population encountered in the clinical routine, contrary to our study population, which also comprises patients with diabetes and likely some with renal failure. While diabetes was not an independent predictor of MBF in our population, renal failure may have confounded our results, potentially resulting in lower MFR compared to completely healthy patients. Furthermore, Sperry’s selection process could have introduced a substantial selection bias in that, for example, older male patients, who are expected to have some coronary calcifications due to natural progression, were excluded from the study. Although comparison of absolute reference MBF values of the Sperry et al. and this study is difficult due to the different tracer properties, it remains noticeable that in this study, sex had a relevant influence on the collected flow values, but age did not have a significant effect. One of the strengths of the present study is the meticulous approach to identifying modifying and potentially competing factors that influence the MBF and assessing their importance. In contrast, Sperry et al. appear to select the two factors age and gender for stratification arbitrarily. It is, therefore, quite possible that unrecognized confounders were present in the Sperry et al. study due to the mentioned potential selection bias. It may seem intuitive that MBF values are dependent not only on gender but also on age. However, while the evidence on the former is relatively well established [21–23], the literature regarding the latter is somewhat controversial. Some studies report a significant decline of sMBF and MFR above 70 years of age [19]; others, a more complex relation between age and sMBF [22]; and yet others do not find a correlation between sMBF or MFR and age [24]. In light of this observation and this study’s results, it may be hypothesized that age is not per se a factor influencing MBF but rather a surrogate marker, reflecting the prevalence of direct modifiers of the coronary vasodilator capacity.

Several studies have previously demonstrated an inverse correlation between BMI and MBF and/or MFR [25, 26]. The results from the present study align with these findings and support the growing recognition of the impact of BMI along with potential metabolic dysregulation and low-grade inflammation on the coronary microvasculature function [27].

In conclusion, the results presented in this study provide novel and comprehensive insights into the interplay between quantitative MBF values as assessed by 13N-ammonia and specific factors that may confound these values. This is important because establishing reference values for MBF and MFR in specific patient scenarios is of great significance for clinical routine and future research. This knowledge may be particularly important for future research as there is a new and growing paradigm in cardiology that stresses the importance of evaluating overall myocardial perfusion as a metric for potential underlying microvascular dysfunction instead of merely focusing on stenoses in epicardial coronary arteries in cardiac risk stratification [5].

### Limitations

Our study has important limitations, including its observational, single-center design. Despite using multivariable-adjusted models, residual confounding cannot be excluded with certainty. Regarding BMI, we ensured that body habitus was not driving the results through an (inadequate) imaging protocol where myocardial counts were related to BMI. However, we cannot confidently exclude that BMI affected image acquisition in some way. However, this does not necessarily reduce the clinical importance of our findings unless such factors or technical limitations of PET MPI in general can be identified and ultimately overcome. The main limitation of the present study, however, is the lack of outcome data. Future studies are needed to evaluate the prognostic value of reference limits for further corroboration and refinement. Another noteworthy limitation is that, due to the retrospective nature of this study, we lack conclusive data on renal function in our patients and, hence, cannot evaluate the potential effect of renal failure on MBF. Although the evidence regarding the effect of renal failure on quantitative MBF is somewhat unclear, with some studies demonstrating a reduced MFR due to increased rMBF and comparable sMBF and others showing comparable MFR, it is not unlikely that renal failure may have confounded our results. Finally, our results are limited to PET MPI with 13N-ammonia, and it was beyond the scope of the current study to assess how the use of different scanners or software solutions may have impacted our results.

## Electronic supplementary material

Below is the link to the electronic supplementary material.

Supplementary Material 1

## Data Availability

The data underlying this manuscript will be shared on reasonable request to the corresponding author.
