# Supplementary material for: Myocardial blood flow reference values for 13N-ammonia PET myocardial perfusion imaging in patients without flow-limiting coronary artery disease
Source: Eur J Nucl Med Mol Imaging. 2025 Mar 14;52(9):3353–63. doi: 10.1007/s00259-025-07196-0 (PMC12222243; doi:10.1007/s00259-025-07196-0)
Supplement: Supplementary file 1 — Supplementary Material 1 [file 259_2025_7196_MOESM1_ESM.docx]

**SUPPLEMENTARY RESULTS**

**Predictive models extended to include cardiac medication**

A supplementary multiple regression analysis was run to identify possible predictors of quantitative MBF measurements and MFR, including also cardiac medication (i.e., antithrombotics, beta-blockers, antihypertensive medication and statins).

For rMBF, the model statistically significantly predicted rMBF F(14,769) = 18.496, adjusted r^2^ = 0.254, p < 0.001). Among all variables, BMI (β = -0.442, p < 0.001, hypertension (β = 0.170, p < 0.001), and sex (β = -0.151, p < 0.001) added independently and statistically significantly to the prediction. Figure S2A provides an overview of the importance of the predictors.

For sMBF, multiple regression analysis, including the same variables, revealed that the model statistically significantly predicted sMBF F(14,769) = 32.053, adjusted r^2^ = 0.376, p < 0.001). Among all variables, only BMI (β = -0.567, p < 0.001) and sex (β = - 0.191, p < 0.001) added independently and statistically significantly to the prediction. Figure S2B provides an overview of the importance of the predictors.

Finally, multiple regression analysis with the same variables for MFR revealed that the model very weakly but statistically significantly predicted MFR F(14,769) = 3.996, adjusted r^2^ = 0.055, p < 0.001). Among all variables, BMI (β = -0.166, p < 0.001) and CACS (β = -0.114, p = 0.003) added independently and statistically significantly to the prediction. Figure S2C provides an overview of the importance of the predictors.

Durban-Watsons statistics for all regression analyses did not indicate any autocorrelation (with values ranging between 1.883 and 2.008), and all tests for multicollinearity were negative.

**SUPPLEMENTARY FIGURES**


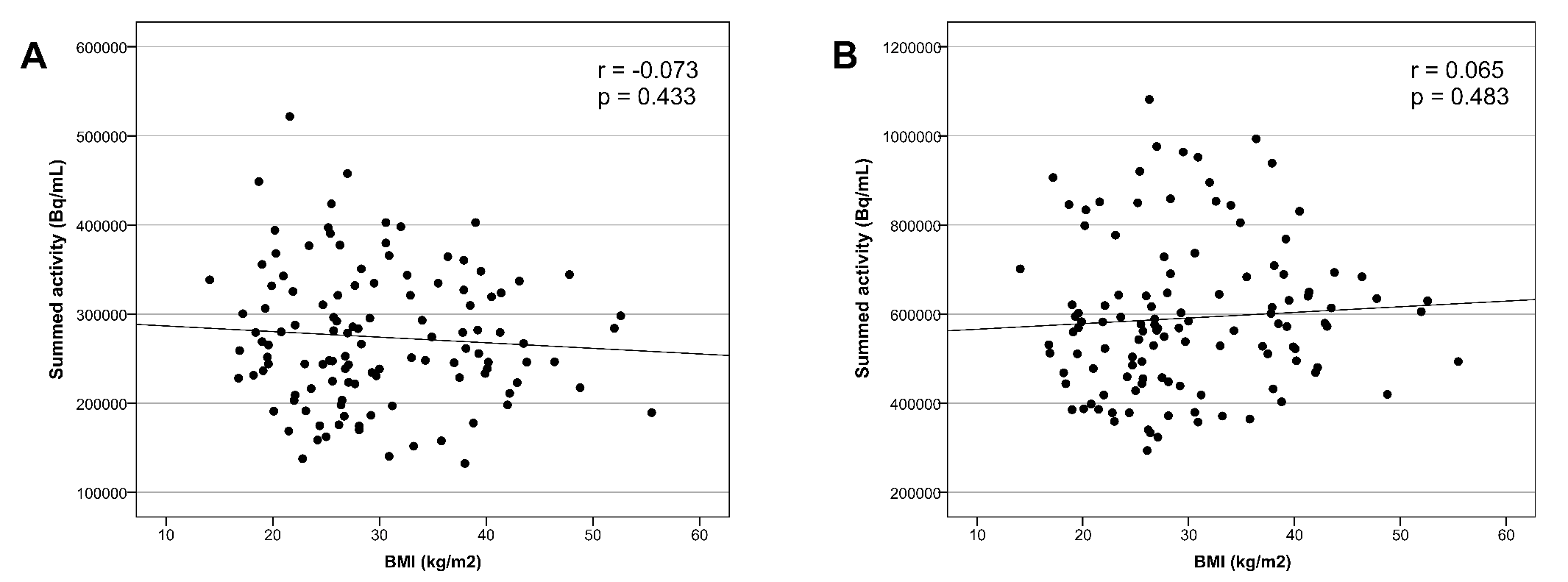


**Fig S1. Correlation between BMI and summed left ventricular myocardium radiotracer activity** during rest (A) and stress (B) dynamic acquisition for 118 randomly selected patients.


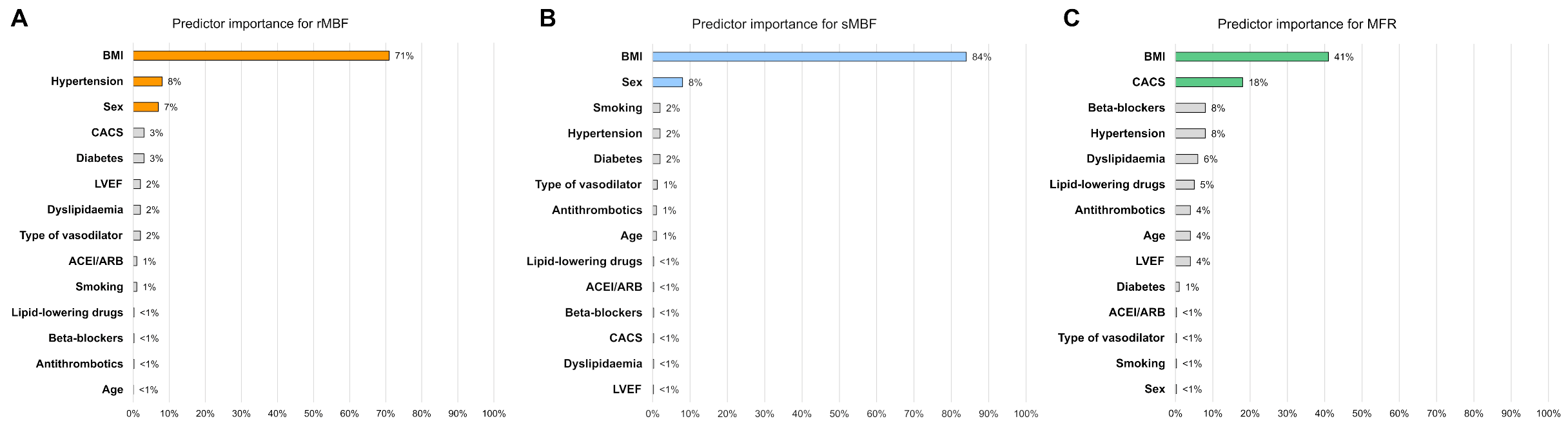


**Fig S2. Predictor importance for MBF.** The relative importance of each predictor in estimating the prediction model for rMBF (A), sMBF (B), and MFR (C) is provided. Statistically non-significant predictors are greyed out.

**SUPPLEMENTARY TABLES**

**Table S1. Uncorrected myocardial flow parameters in the overall population and stratified by sex**

|  |  | Sex | |  |
| --- | --- | --- | --- | --- |
|  | All patients (n=784) | Female (n=315) | Male (n=469) | p-value |
| rMBF_uncorrected_ (ml ∙ min^-1^ ∙ g^-1^)  Lower reference limit  Upper reference limit | 0.80 [0.65-1.01]  0.50  1.52 | 0.88 [0.73-1.09]  0.57  1.59 | 0.75 [0.61-0.95]  0.48  1.46 | **<0.001** |
| sMBF (ml ∙ min^-1^ ∙ g^-1^)  Lower reference limit  Upper reference limit | 2.41 [1.97-2.94]  1.42  3.73 | 2.59 [2.07-3.10]  1.55  3.90 | 2.27 [1.89-2.80]  1.34  3.58 | **<0.001** |
| MFR_uncorrected_  Lower reference limit  Upper reference limit | 2.85 [2.39-3.47]  1.90  4.54 | 2.78 [2.35-3.35]  1.85  4.22 | 2.90 [2.40-3.60]  1.94  4.73 | **0.033** |

Values are median and IQR in brackets and lower (i.e., 5^th^ percentile) and upper (i.e., 95^th^ percentile) reference limits.

**Table S2. Uncorrected myocardial flow parameters stratified by BMI groups**

|  | BMI (kg/m^2^) | | |  |
| --- | --- | --- | --- | --- |
|  | < 25.0 (n=278) | 25.0-29.9 (n=214) | ≥ 30.0 (n=292) | p-value |
| rMBF_uncorrected_ (ml ∙ min^-1^ ∙ g^-1^)  Lower reference limit  Upper reference limit | 0.92 [0.75-1.21]  0.56  1.71 | 0.79 [0.66-1.00] †  0.53  1.43 | 0.74 [0.60-0.87] ‡  0.45  1.20 | **<0.001** |
| sMBF (ml ∙ min^-1^ ∙ g^-1^)  Lower reference limit  Upper reference limit | 2.93 [1.80-3.36]  1.80  4.10 | 2.43 [2.04-2.84] †  1.54  3.68 | 2.03 [1.64-2.39] ^*^‡  1.22  3.04 | **<0.001** |
| MFR_uncorrected_  Lower reference limit  Upper reference limit | 3.02 [2.45-3.71]  1.91  4.88 | 2.95 [2.52-3.56]  2.02  4.42 | 2.70 [2.28-3.18] †‡  1.74  3.98 | **<0.001** |

Values are median and IQR in brackets and lower (i.e., 5^th^ percentile) and upper (i.e., 95^th^ percentile) reference limits.

* p < 0.001 vs. BMI 25.0-29.9 kg/m^2
 †^ p = 0.027 vs. BMI 25.0-29.9 kg/m^2^
 ‡ p < 0.001 vs. BMI <25.0 kg/m^2^

**Table S3. Uncorrected myocardial flow parameters stratified by sex and BMI groups**

|  | Female | | | | Male | | | |
| --- | --- | --- | --- | --- | --- | --- | --- | --- |
|  |  | rMBF_uncorrected_  (ml ∙ min^-1^ ∙ g^-1^) | sMBF  (ml ∙ min^-1^ ∙ g^-1^) | MFR_uncorrected_ |  | rMBF _uncorrected_ (ml ∙ min^-1^ ∙ g^-1^) | sMBF  (ml ∙ min^-1^ ∙ g^-1^) | MFR_uncorrected_ |
| BMI (kg/m^2^) | n |  |  |  | n |  |  |  |
| < 25.0 | 121 | 0.97 [0.82-1.27]  0.65  1.79 | 3.04 [2.60-3.50]  1.86  4.27 | 3.00 [2.46-3.59]  1.95  4.64 | 157 | 0.84 [0.66-1.19]  0.53  1.68 | 2.75 [2.32-3.24]  1.76  3.91 | 3.02 [2.44-3.81]  1.90  5.04 |
| 25.0-29.9 | 61 | 0.91 [0.74-1.13]  0.60  1.55 | 2.65 [2.36-3.03]  1.69  3.91 | 2.78 [2.54-3.40]  1.93  4.08 | 153 | 0.75 [0.60-0.91]  0.51  1.38 | 2.28 [1.99-2.76]  1.51  3.56 | 3.00 [2.50-3.67]  2.03  4.65 |
| ≥ 30.0 | 133 | 0.81 [0.66-0.98]  0.52  1.34 | 2.11 [1.79-2.53]  1.32  3.27 | 2.56 [2.20-3.07]  1.74  4.01 | 159 | 0.67 [0.56-0.80]  0.43  1.12 | 1.90 [1.53-2.20]  1.11  2.87 | 2.75 [2.31-3.30]  1.70  3.98 |

Values are median and IQR in brackets and lower (i.e., 5^th^ percentile) and upper (i.e., 95^th^ percentile) reference limits.

**Table S4. Uncorrected myocardial flow parameters in patients with versus without hypertension in the overall population and stratified by sex**

|  | Overall population | | | Female | | | Male | | |
| --- | --- | --- | --- | --- | --- | --- | --- | --- | --- |
|  | No hypertension (n=384) | Hypertension  (n=400) | p-value | No hypertension (n=156) | Hypertension (n=159) | p-value | No hypertension (n=228) | Hypertension (n=241) | p-value |
| rMBF_uncorrected_  (ml ∙ min^-1^ ∙ g^-1^)  Lower reference limit  Upper reference limit | 0.75 [0.63-0.98]  0.51  1.50 | 0.84 [0.68-1.05]  0.50  1.52 | **0.01** | 0.84 [0.70-1.09]  0.57  1.54 | 0.90 [0.78-1.09]  0.54  1.65 | 0.071 | 0.72 [0.58-0.86]  0.48  1.44 | 0.78 [0.64-1.00]  0.49  1.48 | **0.003** |
| sMBF (ml ∙ min^-1^ ∙ g^-1^)  Lower reference limit  Upper reference limit | 2.51 [2.03-2.99]  1.38  3.77 | 2.30 [1.93-2.91]  1.43  3.68 | **0.016** | 2.68 [2.09-3.13]  1.52  3.92 | 2.52 [2.07-3.08]  1.56  3.90 | 0.403 | 2.39 [1.97-2.85]  1.30  3.68 | 2.15 [1.84-2.77]  1.34  3.52 | **0.011** |
| MFR_uncorrected_  Lower reference limit  Upper reference limit | 2.98 [2.50-3.74]  1.96  4.88 | 2.73 [2.28-3.22]  1.89  3.99 | **<0.001** | 2.85 [2.42-3.52]  1.80  4.61 | 2.73 [2.29-3.20]  1.86  3.89 | **0.048** | 3.14 [2.57-3.81]  2.05  5.00 | 2.72 [2.27-3.27]  1.78  4.05 | **<0.001** |

Values are median and IQR in brackets and lower (i.e., 5^th^ percentile) and upper (i.e., 95^th^ percentile) reference limits.

**Table S5. Uncorrected myocardial flow parameters stratified by CACS groups**

|  | CACS | | | |  |
| --- | --- | --- | --- | --- | --- |
|  | 0 (n=182) | 1-99 (n=246) | 100-399 (n=171) | >400 (n=185) | p-value |
| rMBF_uncorrected_ (ml ∙ min^-1^ ∙ g^-1^)  Lower reference limit  Upper reference limit | 0.75 [0.62-0.97]  0.51  1.60 | 0.79 [0.64-1.00]  0.48  1.57 | 0.82 [0.67-1.02]  0.52  1.40 | 0.84 [0.66-1.05] ^*^  0.51  1.52 | **0.005** |
| sMBF (ml ∙ min^-1^ ∙ g^-1^)  Lower reference limit  Upper reference limit | 2.59 [2.05-3.05]  1.49  3.96 | 2.38 [1.82-2.96] ^*^  1.33  3.57 | 2.38 [2.03-2.92]  1.54  3.90 | 2.29 [1.86-2.78] ^*^  1.36  3.61 | **0.023** |
| MFR_uncorrected_  Lower reference limit  Upper reference limit | 3.15 [2.50-3.91]  1.96  4.92 | 2.80 [2.29-3.50] †  1.80  4.44 | 2.85 [2.49-3.43] ^*^  1.95  4.24 | 2.65 [2.26-3.11] †  1.87  3.87 | **<0.001** |

Values are median and IQR in brackets and lower (i.e., 5^th^ percentile) and upper (i.e., 95^th^ percentile) reference limits.

* p < 0.05 vs. CACS 0
 † p < 0.005 vs. CACS 0

**Table S6. Uncorrected myocardial flow parameters stratified by type of vasodilator stress**

|  | Vasodilator | |  |
| --- | --- | --- | --- |
|  | Adenosine (n=157) | Regadenoson (n=627) | p-value |
| rMBF_uncorrected_ (ml ∙ min^-1^ ∙ g^-1^)  Lower reference limit  Upper reference limit | 0.78 [0.64-1.01]  0.50  1.54 | 0.81 [0.65-1.01]  0.50  1.51 | 0.590 |
| sMBF (ml ∙ min^-1^ ∙ g^-1^)  Lower reference limit  Upper reference limit | 2.55 [2.01-3.09]  1.42  3.83 | 2.38 [1.97-2.92]  1.38  3.67 | 0.068 |
| MFR_uncorrected_  Lower reference limit  Upper reference limit | 3.03 [2.40-3.71]  1.85  5.08 | 2.82 [2.38-3.40]  1.92  4.31 | **0.043** |

Values given are median and IQR in brackets and lower (i.e., 5^th^ percentile) and upper (i.e., 95^th^ percentile) reference limits.

**Table S7. Myocardial flow parameters stratified by age groups**

|  | Age (years) | | | |  |
| --- | --- | --- | --- | --- | --- |
|  | 23-55 (n=194) | 56-65 (n=235) | 66-75 (n=239) | 76-92 (n=116) | p-value |
| rMBF (ml ∙ min^-1^ ∙ g^-1^)  Lower reference limit  Upper reference limit | 0.71 [0.58-0.89]  0.45  1.34 | 0.71 [0.60-0.90]  0.49  1.31 | 0.78 [0.64-0.97] ^*^  0.50  1.29 | 0.84 [0.72-0.98] †^○^  0.55  1.44 | **<0.001** |
| sMBF (ml ∙ min^-1^ ∙ g^-1^)  Lower reference limit  Upper reference limit | 2.40 [1.83-2.98]  1.28  3.65 | 2.38 [1.96-2.91]  1.49  3.76 | 2.36 [2.00-2.89]  1.36  3.66 | 2.54 [2.11-3.04]  1.53  3.92 | 0.127 |
| MFR  Lower reference limit  Upper reference limit | 3.21 [2.56-3.78]  2.12  5.04 | 3.19 [2.64-3.82] †  2.23  4.71 | 3.00 [2.50-3.52] ^*^  2.00  4.53 | 2.97 [2.55-3.52] †^○^  2.16  4.17 | **0.001** |

Values given are median and IQR in brackets as well as lower (i.e., 5^th^ percentile) and upper (i.e., 95^th^ percentile) reference limits.

* p < 0.05 vs. 23-55 years
 † p < 0.005 vs. 23-55 years
 ^○^ p < 0.005 vs. 56-65 years

**Table S8. Coronary vascular resistance in the overall population and stratified by sex**

|  |  | Sex | |  |
| --- | --- | --- | --- | --- |
|  | All patients (n=784) | Female (n=315) | Male (n=469) | p-value |
| rCVR (mmHg ∙ ml^-1^ ∙ min^-1^ ∙ g^-1^)  Lower reference limit  Upper reference limit | 127 [100-155]  70  204 | 114 [96-142]  69  186 | 133 [107-163]  70  213 | **<0.001** |
| sCVR (mmHg ∙ ml^-1^ ∙ min^-1^ ∙ g^-1^)  Lower reference limit  Upper reference limit | 39 [31-48]  24  66 | 36 [29-45]  23  60 | 40 [32-49]  25  70 | **<0.001** |

Values are median and IQR in brackets and lower (i.e., 5^th^ percentile) and upper (i.e., 95^th^ percentile) reference limits.
